# Impact of functional MRI data preprocessing pipeline on default-mode network detectability in patients with disorders of consciousness

**DOI:** 10.3389/fninf.2013.00016

**Published:** 2013-08-22

**Authors:** Adrian Andronache, Cristina Rosazza, Davide Sattin, Matilde Leonardi, Ludovico D'Incerti, Ludovico Minati, on behalf of the Coma Research Centre (CRC) – Besta Institute

**Affiliations:** ^1^Neuroradiology Unit, Fondazione IRCCS Istituto Neurologico “Carlo Besta”Milan, Italy; ^2^Scientific Department, Fondazione IRCCS Istituto Neurologico “Carlo Besta”Milan, Italy; ^3^Neurology, Public Health, Disability Unit, Scientific Department, Fondazione IRCCS Istituto Neurologico “Carlo Besta”Milan, Italy

**Keywords:** functional MRI (fMRI), resting-state, functional connectivity, disorders of consciousness, vegetative state, minimally-conscious state, data preprocessing

## Abstract

An emerging application of resting-state functional MRI (rs-fMRI) is the study of patients with disorders of consciousness (DoC), where integrity of default-mode network (DMN) activity is associated to the clinical level of preservation of consciousness. Due to the inherent inability to follow verbal instructions, arousal induced by scanning noise and postural pain, these patients tend to exhibit substantial levels of movement. This results in spurious, non-neural fluctuations of the rs-fMRI signal, which impair the evaluation of residual functional connectivity. Here, the effect of data preprocessing choices on the detectability of the DMN was systematically evaluated in a representative cohort of 30 clinically and etiologically heterogeneous DoC patients and 33 healthy controls. Starting from a standard preprocessing pipeline, additional steps were gradually inserted, namely band-pass filtering (BPF), removal of co-variance with the movement vectors, removal of co-variance with the global brain parenchyma signal, rejection of realignment outlier volumes and ventricle masking. Both independent-component analysis (ICA) and seed-based analysis (SBA) were performed, and DMN detectability was assessed quantitatively as well as visually. The results of the present study strongly show that the detection of DMN activity in the sub-optimal fMRI series acquired on DoC patients is contingent on the use of adequate filtering steps. ICA and SBA are differently affected but give convergent findings for high-grade preprocessing. We propose that future studies in this area should adopt the described preprocessing procedures as a minimum standard to reduce the probability of wrongly inferring that DMN activity is absent.

## Introduction

In recent years, resting-state functional MRI (rs-fMRI) has attracted substantial research and clinical interest. In contrast with fMRI based on active tasks, it is a straightforward form of functional imaging suitable for the study of patients who are unable to follow procedural instructions or are generally unresponsive. Alongside practical considerations, there is increased awareness of the importance of intrinsic brain activity in supporting behavioral function and determining metabolism (e.g., Fox and Raichle, 2007; Rosazza and Minati, 2011).

An emerging application of rs-fMRI is the study of patients with disorders of consciousness (DoC), an etiologically heterogeneous condition that typically follows substantial brain damage due to vascular, hypoxic or traumatic insults. The clinical phenotype is highly variable, ranging from complete absence of wilful responses (vegetative state) to situations where awareness is fluctuating and a rudimentary communication code may be established (minimally-conscious state or severe disability; e.g., Laureys, 2005; Owen and Coleman, 2008).

In the healthy brain, rs-fMRI reveals a set of well-reproducible, separable activity components which appear to correlate with specific sensory, motor and cognitive functions (Biswal et al., 2010; Allen et al., 2011). In particular, the default-mode network (DMN) has received considerable attention as a potential proxy of large-scale integrative processes related to awareness, interoception and memory consolidation. This bi-hemispheric network has its main constituent nodes in the precuneus, lateral parietal cortex and medial prefrontal cortex, and exhibits a well-reproducible, graded response to wakefulness, sleep and coma (Raichle et al., 2001; Buckner et al., 2008; Rosazza and Minati, 2011).

The severity of the clinical phenotype of patients in vegetative state or minimally conscious state is reflected in the level of residual functional connectivity across the DMN (Boly et al., 2009; Cauda et al., 2009; Vanhaudenhuyse et al., 2010; Soddu et al., 2011) and other networks (Owen et al., 2006; Owen and Coleman, 2007), with recent work also indicating specific alterations in the relationships across networks, particularly between the DMN and the fronto-parietal component (Boly et al., 2009; Noirhomme et al., 2010). Importantly, the intensity of connectivity across the DMN nodes and, consequentially, the detectability of the network as a whole appear to be coupled to the level of residual consciousness, as assessed by established clinical scales (Vanhaudenhuyse et al., 2010). Rs-fMRI is therefore of particular relevance for the study of DoC patients, since it can help to determine how large-scale integrative processes are affected in the presence of impaired consciousness.

A major challenge for the use of rs-fMRI in clinical populations is head movement, consequential to several factors including the inability for the patient to understand and comply with verbal instructions, emotional arousal caused by the scanner environment, decorticate or decerebrate posture and postural pain. Even when gross imaging artifacts are absent owing to the rapidity of echo-planar acquisition and time-series volumes are accurately realigned, significant signal modulations are introduced by movement due to multiple factors including inhomogeneous coil sensitivity, inhomogeneous coil loading by the head, interaction between susceptibility gradients and head movement, and partial-volume effects. Such contaminations can introduce spurious correlations as well as mask coherent neuronal sources of blood-oxygen level-dependent (BOLD) signal fluctuations, making it impossible to draw reliable inferences on the degree of preservation of functional connectivity (Friston et al., 1996; Hutton et al., 2002; Johnstone et al., 2006; Strother, 2006; Power et al., 2012).

Thus, head movement represents a particularly insidious confound for the study of patients with DoC (Giacino et al., 2006; Owen and Coleman, 2007; Soddu et al., 2011) because very large variability of residual neuronal function is expected ab-initio, as testified by the fact that the EEG can range from near-normal to near-isoelectric (Soddu et al., 2012), and operators may therefore be inclined to accept the findings of rs-fMRI uncritically.

Pre-processing techniques to remove physiological noise and movement artifacts in rs-fMRI have been investigated extensively in healthy participants with reference to both data-driven (i.e., independent-component analysis, ICA) and anatomy-driven (i.e., seed-based analysis, SBA) analyses (Birn et al., 2006; Lund et al., 2006; Fox et al., 2009; Murphy et al., 2009; Weissenbacher et al., 2009; Van Dijk et al., 2010). Existing studies have demonstrated the importance of removing by linear regression co-variance with movement parameters (Power et al., 2012; Van Dijk et al., 2012) and physiological variables (Corfield et al., 2001; Birn et al., 2006; Weissenbacher et al., 2009), either measured directly or inferred from the rs-fMRI time-series. Several reports have also underlined the utility of removing diffuse and un-specific signal fluctuations, indexed by averaging signal over the whole brain: while this may induce artifactual anti-correlations, it strongly limits the effect of unaccounted sources of global noise over inter-regional correlation estimates (Desjardins et al., 2001; Macey et al., 2004; Murphy et al., 2009). Further, it has been demonstrated that the confounding effect of movement can be attenuated by combining regression of the movement parameters with the exclusion and replacement by interpolation of selected contaminated volumes; this approach is particularly appropriate in the presence of brief, large movements (Carp, 2013). In recent work on healthy controls, the effect of the available filtering techniques was systematically investigated, and it was concluded that consideration of the parameters listed above alongside the first temporal derivative of movement enhances the sensitivity and stability of connectivity inferences (Van Dijk et al., 2012; Satterthwaite et al., 2013). There is, however, a lack of consistency in terms of preprocessing methods across the existing rs-fMRI investigations of residual neural function in DoC: while in some studies movement-related, physiological and unspecific global BOLD signal variance were explicitly removed, in others more basic data-preprocessing chains were utilized. In particular, none of studies the authors are aware of have included specific preprocessing steps to reduce the impact of the large, sudden movements and substantial gross anatomical damage present in this population, e.g., by outlier rejection and masking (Boly et al., 2009; Cauda et al., 2009; Vanhaudenhuyse et al., 2010; Soddu et al., 2011, 2012).

A crucial and unresolved question pertains to what extent the large variability observed in this clinical group truly represents neural differences rather than being consequential to movement and other confounds. From a methodological viewpoint, there is a need for a systematic evaluation of the effect of preprocessing choices on data from this specific clinical population, and for clear guidelines on how to best preprocess the rs-fMRI datasets acquired for diagnostic and research purposes, to ensure the best yield in terms of detectability of residual DMN function.

Here, we comprehensively investigated how inserting specific filtering steps in the preprocessing chain can improve the detectability of the DMN or its residual portions in a clinically and aetiologically heterogeneous population of DoC patients. We hypothesized that using a tailored preprocessing chain would substantially improve DMN detectability, as revealed by automated measurements as well as qualitative assessments. Since ICA and SBA are often interchangeably utilized, in spite of their substantially different computational properties (e.g., Rosazza et al., 2012), we also investigated whether the two techniques are differently sensitive to data preprocessing.

## Methods

### Participants

All investigational protocols were approved by the institutional ethics committee and written informed consent was always obtained from the healthy participants and the legal representative of the patients. The study was conducted on 30 consecutive patients with a clinical diagnosis of vegetative state or minimally conscious state and 33 healthy volunteers. The selection criterion for the patients was the detectability of the DMN with at least one data analysis technique in one of the 5 different procedures; patients in whom in the DMN appeared completely absent, irrespective of data preprocessing and analysis choices, were excluded a-priori, since the purpose of the present study was to demonstrate the differential effect of data preprocessing choices on DMN detectability. In the recruitment period, 25 other patients were scanned, but rejected as the DMN was not detectable with either ICA or SBA, irrespective of preprocessing.

The average patient age was 54 years (range 22–82), average disease duration was 34 months (range 7–105), 13 patients were female; regarding etiology, 13 have had head trauma, 11 intracranial hemorrhage and 6 cerebral anoxia. For controls, the average age was 39 years (range 17–66). All patients were assessed and evaluated with the Coma Recovery Scale-Revised (CRS-R; Giacino et al., 2004; Lombardi et al., 2007) and with the Coma Near-Coma scale (CNC; Rappaport, 2005). According to internationally accepted criteria (Multi-Society, 1994; Giacino, 2004), 15 patients were diagnosed as being in vegetative state (CRS-R 6.4 ± 1.8, CNC 2.4 ± 0.5) with the remaining 15 being in minimally conscious state (CRS-R 13.7 ± 5.4, CNC 1.3 ± 0.6).

### Data acquisition

Functional imaging was performed on a 3 Tesla scanner equipped with a 32-channel head coil (Achieva, Philips Healthcare BV, Best, NL). Two hundred functional volumes were acquired by means of an axial gradient-echo echo-planar sequence, having *TR* = 2800 ms, *TE* = 30 ms, α = 70°, 2.5 mm isotropic voxel size, 90 × 95 matrix size, 50 slices with 10% gap, ascending order. Sequence duration was ~9.5 min. When possible given the patient posture, the head was gently restrained using foam pillows, and a wedge was positioned under the knees to minimize spine movement. The relatively small voxel size was chosen primarily to reduce spatial distortions in the presence of inhomogeneous susceptibility due to macroscopic lesions and deposits.

### Data preprocessing

**Figure 1:**
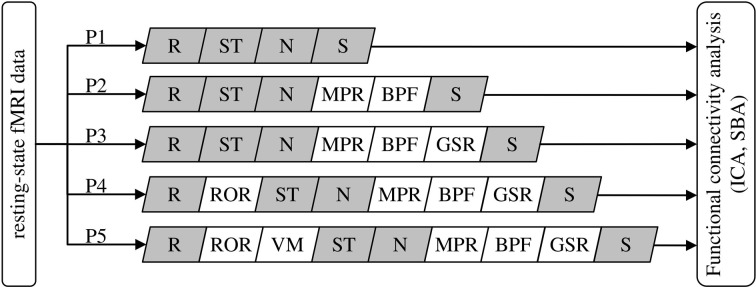
**Definition of the data preprocessing pipeline for the five procedures (P1–P5) under comparison.** R, realignment; ST, slice-timing correction; N, normalization to MNI space; S, spatial smoothing; MPR, removal of co-variance with movement parameters; BPF, band-pass filtering; GSR, removal of global parenchymal signal; ROR, removal of realignment outliers; VM, ventricle masking. The modules in gray are standard SPM8 functions, the others are functions developed in-house (see text).

Five data preprocessing procedures of increasing complexity, consisting of different combinations of standard modules implemented in SPM8 (Wellcome Trust Centre for Neuroimaging, London, UK) and custom code developed in MatLab 7 (Mathworks Inc., Natick MA, USA), were compared (Figure 1).

Procedure 1 (P1) consisted of the standard SPM8 workflow for fMRI: rigid-body realignment to average volume with minimization of squared differences (R), slice-timing correction (ST), normalization to MNI space by co-registration to the individual *T*_1_ structural scan and subsequent segmentation (N), and spatial smoothing using an isotropic Gaussian kernel having FWHM 8 mm (S). The absence of gross normalization errors was visually confirmed by an experienced operator for all patients.

Procedure 2 (P2) additionally included masking with a standard brain-mask to remove all voxels outside the brain outline (but not the ventricles), removal of movement-related variance (MPR) and band-pass filtering (BPF). Movement-related variance was removed by multilinear regression of the individual voxel time-courses with respect to the six movement vectors, measured in absolute terms with respect to the first volume. BPF was performed removing baseline fluctuations, e.g., related to gradient system heating, by fitting and subtracting a 3rd order polynomial, followed by low-pass filtering with a Butterworth filter of order 1 having *f*_−3*dB*_= 0.1 Hz and applied twice in opposite directions to attenuate rapid, non-neural BOLD signal fluctuations (e.g., cardiac pulsatility).

Procedure 3 (P3) added global signal regression (GSR), i.e., the removal by linear regression of the variance correlated to average signal intensity time-course calculated over all voxels included in the brain parenchyma mask, derived from SPM segmentation. Performing this operation is advised in Weissenbacher et al. (2009) and Van Dijk et al. (2010) as it attenuates topographically-unspecific temporal variance, e.g., related to residual baseline instability effects and systemic sources of physiological noise, which can positively bias connectivity inferences. While this step is generally deemed not necessary for ICA, here a common set of preprocessing pipelines was considered and ICA/SBA were therefore performed on the same data, including the GSR step.

Procedure 4 (P4) added the removal of realignment outliers (ROR), i.e., the identification and replacement of volumes having large residual mean-square difference from the average volume after realignment, indicating the presence of macroscopic imaging artifacts due to head movement, similarly to the work of Carp (2013). Consideration of mean-square signal difference enables a more direct assessment of signal contamination with respect to distance from reference volume; for example, in presence of sudden movements this criterion promptly identifies volumes affected by “shear” between the first and last sections: for these, the translation/rotation realignment parameters may not differ substantially from the neighboring volumes but the attained overlap with the reference volume is poor due to distortion. Volumes having residual mean-square difference larger than 1.5 times the interquartile range calculated over all volumes of a series were considered potential outliers. However, outlier rejection was actually performed only if the mean-square difference exceeded a reference value, empirically set to 10% of the average of the three mean-square differences obtained by artificially displacing by one voxel along the three axes the image used as reference in the realignment process. When less than 10 consecutive outliers were identified, to avoid introducing temporal discontinuities they were replaced with the multilinear interpolation of the nearest preserved volumes; groups of more than 10 consecutive outliers were removed altogether.

Procedure 5 (P5) additionally included masking to remove the ventricles (VM) and was motivated by the observation of very large ventricles with substantial flow-induced signal fluctuations in some patients. For each scan, the ventricles were identified by average signal intensity thresholding followed by morphological filtering to remove speckles, fill holes and identify the connected-component representing the ventricles.

Removal of voxels outside the brain outline (P2) and in the ventricles (P5) was deemed relevant here because it excludes a range of non-neural signal sources such as pulsating cerebrospinal fluid and eye movements; while in healthy participants these do not impact ICA substantially, it was hypothesized that in patients their removal might facilitate the proper un-mixing of weak neural signals, especially given that substantial brain atrophy can be present and the relative representation of cerebrospinal fluid voxels can be substantially higher with respect to controls.

### Data analysis

ICA was performed independently for each participant, using the group ICA of fMRI toolbox (GIFT, MIALab, University of New Mexico, USA) and assuming a fixed number of 20 independent components (Calhoun et al., 2001, 2008). The component corresponding to DMN activity was identified upon agreement of two experienced observers, who searched for significant correlation clusters (at *z* > 2) specifically in the precuneus (PCC), lateral parietal (LP), and medial prefrontal regions (MPFC) and considered the specificity of correlation in such regions with respect to the rest of the brain. A component was deemed a candidate DMN if it exhibited focal activity in at least two regions. In the rare instances where DMN activity appeared “split” between two hemispheric components (see results), the components were merged using the voxel-wise maximum operator before further evaluation.

SBA of the DMN was implemented by extracting two reference time-courses from the average of all voxels in the left and right PCC as defined below, and entering them as regressors in a first-level general-linear model analysis (Fox et al., 2005). For the purpose of the evaluations described below, the maps derived from the two hemispheric regressors were always combined using voxel-wise maximum operator, thresholded and considered together.

To obtain a further measure of intra- and inter-hemispheric connectivity across the DMN nodes, linear regressions were performed between the average BOLD signal time-courses in the left and right PCC, LP, and MPFC.

### Default-mode network evaluation

The detectability of the DMN was evaluated quantitatively with ICA as well as SBA for each preprocessing procedure P1–P5. In order to represent the intensity of DMN activity in the PCC, LP and MPFC, the peak *z*-score was calculated in the corresponding binary masks, obtained by intersecting the corresponding regions of the automated anatomical labeling atlas (AAL; Tzourio-Mazoyer et al., 2002) with the thresholded group-level DMN component maps from the controls, and dilated by 5 voxels to account for potential normalization imperfections. Due to their medial location, the left/right PCC and MPFC ROIs were contiguous; hence they were merged, yielding peak *z*-scores for bilateral PCC, bilateral MPFC, left LP (LP_L_) and right LP (LP_R_). To obtain a measure of correlation specificity, we additionally determined the extent of correlations outside the DMN regions by counting the voxels with a *z*-score > 2 and represented it as percentage of brain volume.

The presence of correlated activity in the DMN nodes on the ICA component map for the DMN was also visually rated by two experienced observers, blinded to participant information and preprocessing procedure, along the following scale: 0—definitely absent, 1—uncertain, 2—definitely present. The scores given by the two observers were averaged together; for patients the inter-rater agreement was 73%, 86%, 91%, and 88% for the PCC, MPFC, LP_L_, and LP_R_ nodes. A global “DMN detectability” score was thereafter calculated summing the scores of the four nodes, and normalized within each patient with respect to the highest score attained individually; this step was introduced to reduce variability related to inter-participant differences, as the interest here was to compare the preprocessing procedures within each case.

### Statistical analysis

For all measures of interest a non-parametric related-samples Friedman test was performed, followed where appropriate by pair-wise Wilcoxon rank tests. Non-parametric tests were chosen in place of ANOVA since some distributions were significantly skewed. To account for multiple comparisons, all *p*-values were corrected using Bonferroni-Holm's procedure (Holm, 1979), performed over all Friedman and Wilcoxon tests, separately for patients and controls. To remove potential bias, scores corresponding to regions where the brain parenchyma was absent due to large anatomical lesions were removed and treated as missing values (10, 3, and 4 instances for the LP_R_, LP_L_, and MPFC, respectively) and imputed to the group median.

## Results

As indicated in Table 1, outlier volumes after rigid-body realignment were detected and rejected for 21 patients (70%) and 5 controls (15%). The results of statistical analyses are given in Tables 2, 3.

**Table 1:** **Statistics on the rejection of realignment-outlier volumes**.

**Any volume rejected?**	**Patients or controls**	**Number of subjects**	**Outliers detected**	**Volumes removed**	**Initial displacement (mm)**		**Residual displacement (mm)**	
					**Average**	**Worst**	**Average**	**Worst**
No	Patients	9	0	0	0.06 ± 0.05	0.15 ± 0.13	0.06 ± 0.05	0.15 ± 0.13
Yes	Patients	21	20 ± 16	16 ± 23	0.13 ± 0.51	0.45 ± 1.85	0.12 ± 0.16	0.41 ± 0.44
Total	Patients	30	13 ± 16	10 ± 20	0.11 ± 0.35	0.45 ± 1.85	0.10 ± 0.12	0.41 ± 0.44
No	Controls	28	0	0	0.05 ± 0.04	0.11 ± 0.12	0.05 ± 0.04	0.11 ± 0.12
Yes	Controls	5	10 ± 7	0	0.08 ± 0.10	0.14 ± 0.17	0.08 ± 0.08	0.13 + 0.10
Total	Controls	33	1 ± 5	0	0.05 ± 0.05	0.14 ± 0.17	0.05 ± 0.05	0.13 + 0.10

In the subjects for whom outliers were detected, the residual displacement after rigid-body realignment of the time-series was reduced by outlier rejection. As described in-text, depending on the presence of contiguous outliers, replacement by interpolation or outright removal was performed. All values are given in mm as mean ± standard deviation of relative displacement between consecutive EPI volumes.

**Table 2:** **Statistical evaluation of the effect of the preprocessing procedures for healthy controls**.

**Analysis method**	**Parameter**	**Main effect (Friedman test)**		***Post-hoc* (Wilcoxon signed ranks test)**			
				**P1–P2**	**P2–P3**	**P3–P4**	**P4–P5**
		**χ^2^**	***p***	***p***	***p***	***P***	***p***
Independent component analysis	Qualitative	8	1	–	–	–	–
	PCC: *z*-score	22	0.007	1	1	0.004	0.001
	MPFC: *z*-score	31	0.0001	0.3	0.03	0.005	0.1
	LP_R_: *z*-score	17	0.07	–	–	–	–
	LP_L_: *z*-score	23	0.006	1	0.6	0.0003	0.02
	Extra-DMN: %	39	<0.0001	0.6	0.02	0.004	0.1
Seed-based analysis	MPFC: *z*-score	71	<0.0001	0.003	0.0002	1	0.3
	LP_R_: *z*-score	73	<0.0001	0.8	0.0005	1	1
	LP_L_: *z*-score	65	<0.0001	1	0.0005	1	1
	Extra-DMN: %	120	<0.0001	<0.0001	<0.0001	0.0001	0.0001
Linear regression	LP_L_-LP_R_: r	100	<0.0001	0.002	<0.0001	0.03	0.03
	PCC_L_-LP_L_: r	112	<0.0001	<0.0001	<0.0001	0.2	0.6
	PCC_R_-LP_R_: r	104	<0.0001	0.0005	<0.0001	1	1
	PCC_L_-MPFC_L_: r	108	<0.0001	<0.0001	<0.0001	0.2	0.01
	PCC_R_-MPFC_R_: r	110	<0.0001	<0.0001	<0.0001	0.08	0.005

As described in-text, Friedman tests followed, where appropriate, by Wilcoxon post-hocs were performed. All p-values are reported following Bonferroni-Holm correction. PCC, posterior cingulate and precuneus; LP, lateral parietal cortex; MPFC, anterior cingulate and medial prefrontal cortex. Subscripts “L” or “R” stand for left or right hemisphere. “Extra-DMN” refers to the proportion of activated voxels outside the expected DMN localization. The effect of preprocessing “grade” was significant for most parameters, with the greatest overall differences being observed between P1–P2 and P2–P3, corresponding to the addition of band-pass filtering and removal of co-variance with the movement vectors and global brain parenchyma signal. See text for details.

**Table 3:** **Statistical evaluation of the effect of the preprocessing procedures for patients**.

**Analysis method**	**Parameter**	**Main effect (Friedman test)**		***Post-hoc* (Wilcoxon signed ranks test)**			
				**P1–P2**	**P2–P3**	**P3–P4**	**P4–P5**
		**χ^2^**	***p***	***p***	***p***	***P***	***p***
Independent component analysis	Qualitative	43	<0.0001	0.01	1	1	1
	PCC: *z*-score	31	0.0002	0.008	1	1	1
	MPFC: *z*-score	19	0.04	0.4	0.04	1	1
	LP_R_: *z*-score	11	0.9	–	–	–	–
	LP_L_: *z*-score	14	0.3	–	–	–	–
	Extra-DMN: %	48	<0.0001	0.002	0.02	1	1
Seed-based analysis	MPFC: *z*-score	49	<0.0001	1	0.002	1	0.8
	LP_R_: *z*-score	58	< 0.0001	0.4	0.01	1	1
	LP_L_: *z*-score	49	< 0.0001	0.4	0.006	1	0.9
	Extra-DMN: %	102	<0.0001	0.0002	0.0002	0.005	0.01
Linear regression	LP_L_-LP_R_: r	66	<0.0001	0.001	0.01	0.9	1
	PCC_L_-LP_L_: r	50	<0.0001	0.014	0.001	1	1
	PCC_R_-LP_R_: r	56	<0.0001	0.004	0.004	1	1
	PCC_L_-MPFC_L_: r	37	<0.0001	0.006	0.0008	1	0.1
	PCC_R_-MPFC_R_: r	37	<0.0001	0.003	0.0006	1	1

As described in-text, Friedman tests followed, where appropriate, by Wilcoxon post-hocs were performed. All p-values are reported following Bonferroni-Holm correction. PCC, posterior cingulate and precuneus; LP, lateral parietal cortex; MPFC, anterior cingulate and medial prefrontal cortex. Subscripts “L” or “R” stand for left or right hemisphere. “Extra-DMN” refers to the proportion of activated voxels outside the expected DMN localization. The effect of preprocessing “grade” was significant for most parameters, with the greatest overall differences being observed between P1–P2 and P2–P3, corresponding to the addition of band-pass filtering and removal of co-variance with the movement vectors and global brain parenchyma signal. See text for details.

**Figure 2:**
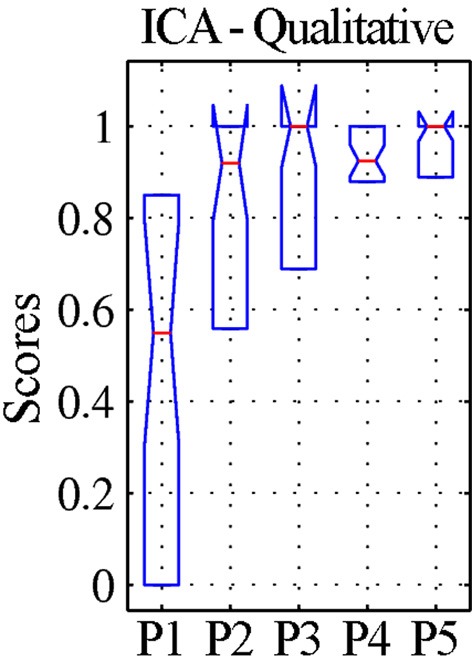
**Qualitative evaluation of the detectability of the whole default-mode network (DMN) extracted by ICA for patients.** The values represent visual assessment scores for activity across the four main nodes (PCC, precuneus; LP_R_, right lateral parietal cortex; LP_L_, left lateral parietal cortex; MPFC, medial prefrontal cortex), averaged between the two raters and normalized within each patient, so that the maximum score of 1 corresponds to the best qualitative appearance of the DMN observed for each case (see text). The box-plots represent the median and inter-quartile ranges of the visual assessment scores. As preprocessing steps were added to the pipeline (P1–P5), the dispersion diminished and the median approached unity, confirming that the DMN was best identifiable after preprocessing using procedure P5.

**Figure 3:**
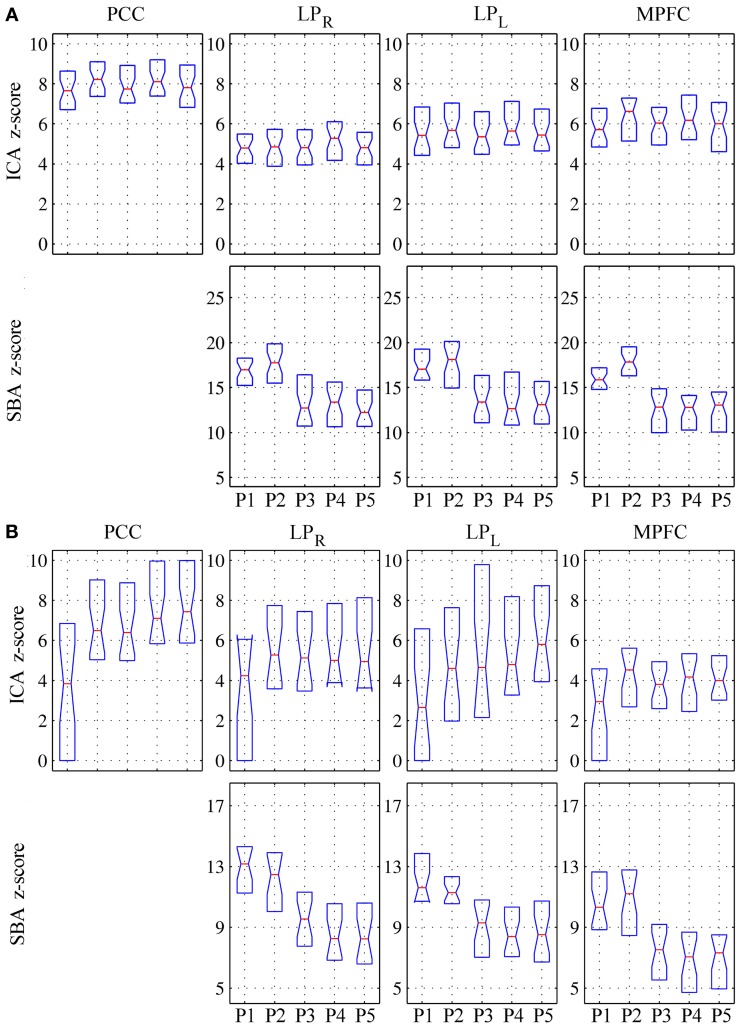
**Peak *z*-scores for activity within the four main DMN nodes for (A) healthy controls and (B) patients.** Top row: DMN component extracted by ICA; bottom row: correlation maps computed using precuneus seeds (SBA). As preprocessing steps were added to the pipeline (P1–P5), the median *z*-scores for the DMN component extracted through ICA generally increased, indicating better component extraction, whereas the *z*-scores from SBA diminished (see text for comment and Figures 6–9).

**Figure 4:**
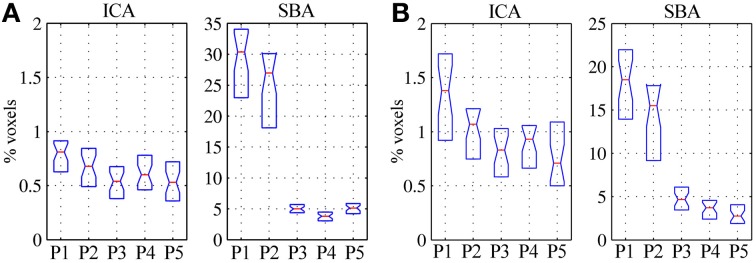
**Volume of significant activations (z = 2) outside the regions-of-interest covering the expected DMN nodes (i.e., PCC, LP_R_, LP_L_, and MPFC), for the DMN maps extracted through ICA (left) and SBA (right), expressed as percent with respect to the parenchymal volume for (A) healthy controls and (B) patients.** As preprocessing steps were added to the pipeline (P1–P5), the extent of activations outside the expected localization of the DMN was progressively reduced, representing greater specificity of the functional connectivity maps; the effect was considerably more marked for SBA than ICA.

In patients, for ICA assessed qualitatively elevating preprocessing grade increased between procedures P1 and P5 the number of patients for whom activity in each node was identifiable (Table 4); at group level, there was a significant difference between procedures P1–P2 only (Figure 2, Table 3). Elevating preprocessing grade also increased the peak correlation scores in the PCC and MPFC, with significant differences between procedures P1–P3 (Figure 3B, Table 3). Alongside improvement of correlation intensity, a reduction in the extent of spuriously correlated activity outside the DMN nodes was also observed, with significant difference between procedures P1–P3 (Figure 4B, Table 3). By contrast, in controls elevating preprocessing grade had no relevant effect on DMN detectability as assessed qualitatively (graph not shown). As reported in Table 2, elevating preprocessing grade nevertheless increased the peak correlation scores, but in this case the differences were primarily observed between procedures P3–P4 (Figure 3A); similar improvements were also detected for extra-DMN correlations (Figure 4A). While the levels of statistical significance of the effect of preprocessing were overall similar between patients and controls, it should be noted that the median magnitudes of the effect of preprocessing and inter-individual variability were substantially larger for patients (Figures 3A vs. 3B and 4A vs. 4B).

**Table 4:** **Qualitative evaluation of default-mode network (DMN) node detectability on the component extracted by ICA**.

**Preprocessing procedure**	**Precuneus (PCC, 30 pts.) (%)**	**Right lateral partietal cortex (LP_R_, 20 pts.) (%)**	**Left lateral parietal cortex (LP_L_, 27 pts.) (%)**	**Medial prefrontal cortex (MPFC, 26 pts.) (%)**
P1	14 (47)	12 (60)	9 (33)	8 (31)
P2	22 (73)	14 (70)	16 (59)	15 (58)
P3	23 (77)	16 (80)	17 (63)	14 (54)
P4	26 (87)	17 (85)	19 (70)	14 (54)
P5	27 (90)	16 (80)	20 (74)	14 (54)

The values represent the number and percentage of patients for whom activity was rated as definitely present. Percentages are adjusted to account for the number of cases where a node was affected by macroscopic anatomical damage (see Methods).

For SBA, in patients elevating preprocessing grade markedly reduced the peak correlation scores across all regions, with a significant difference between procedures P2 and P3 for all regions (Figure 3B, Table 3). A strong reduction in the extent of spuriously correlated activity outside the DMN nodes was also apparent, with significant differences between all procedures (Figure 4B, Table 3). Similar effects were observed in controls (Figures 3A, 4A).

**Figure 5:**
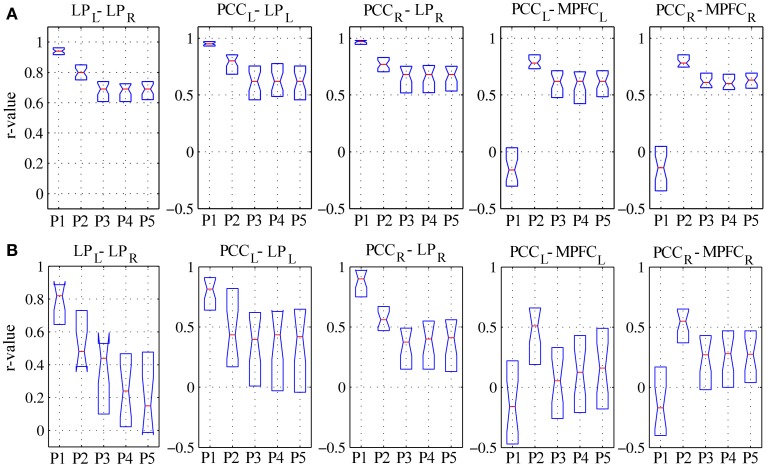
**Linear correlation coefficients for regionally-averaged BOLD signal time-series between DMN regions for (A) healthy controls and (B) patients.** See text for description of the results.

For linear regression between average time-courses of the DMN nodes, preprocessing grade had a significant effect on all pairs investigated. In patients, the linear correlation coefficient between LP_L_-LP_R_, PCC_L_-LP_L_, and PCC_R_-LP_R_ monotonically decreased, whereas that between PCC_L_-MPFC_L_ and PCC_R_-MPFC_R_ displayed a more complex response, overall slightly increasing with disproportionately large values observed for procedure P2; here, significant *post-hoc* differences were found between procedures P1–P3 (Figure 5B, Table 3). The effect of preprocessing grade was more statistically significant in controls than patients primarily owing to substantially smaller inter-individual variability. However, in absolute terms, the difference between P1 and P5 was larger in patients than controls for LP_L_-LP_R_, PCC_L_-LP_L_, and PCC_R_-LP_R_: while elevating preprocessing grade reduced median *r*-values down to about 0.7 in controls (Figure 5A), in patients removal of spurious signal reduced the median *r*-values to below 0.5, and for LP_L_-LP_R_ even below 0.2 (Figure 5B, Table 2). For PCC_L_-MPFC_L_ and PCC_R_-MPFC_R_, there was a converse pattern, wherein elevating preprocessing grade increased correlation much more for controls (up to about 0.6) than patients (about 0.3), plausibly representing the different effects of signal contamination on the anterior-posterior axis and poor preservation of MPFC connectivity in patients (Figures 5A,B, Tables 2, 3).

**Figure 6:**
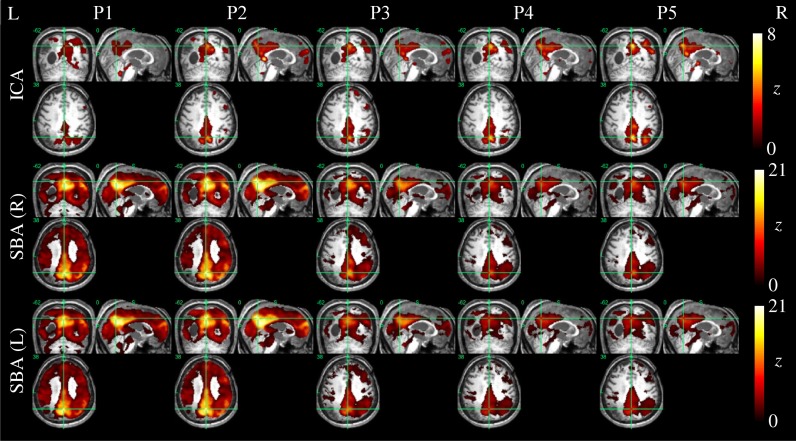
**DMN functional connectivity maps computed with ICA (top row) and SBA (middle and bottom rows) for a patient with a clinical diagnosis of vegetative state (maximum displacement 4.0 mm, 27/200 outlier volumes).** As preprocessing steps were added (left to right, P1–P5), activity in the right angular gyrus became more evident on the ICA maps. For SBA, enhanced preprocessing had the effect of progressively reducing the diffuse correlations observed throughout the brain, revealing a topographical pattern that converged to that extracted by ICA.

**Figure 7:**
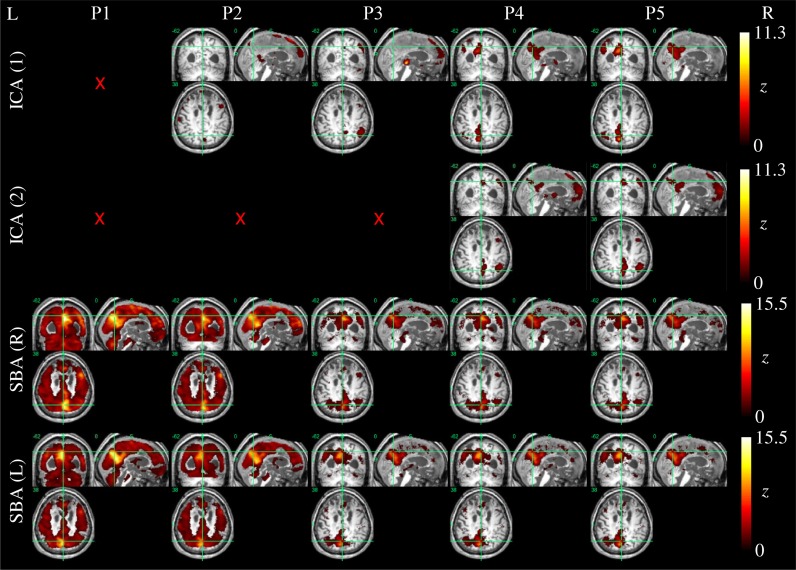
**DMN functional connectivity maps computed with ICA (top row) and SBA (middle and bottom rows) for a patient with a clinical diagnosis of vegetative state (maximum displacement 0.8 mm, 3/200 outlier volumes); red crosses denote inability to identify DMN activity in any of the 20 components extracted by ICA.** As preprocessing grade was elevated (left to right, P1–P5), coherent activity between the precuneus and the angular gyri became identifiable through ICA and SBA. Notably, in this patient an apparent “split” between left and right DMN connectivity was observed through both analyses.

**Figure 8:**
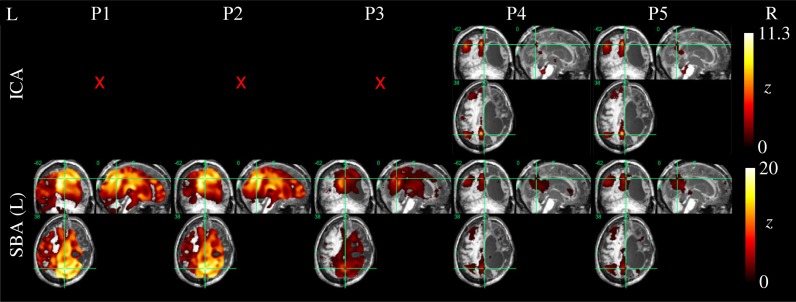
**DMN functional connectivity maps computed with ICA (top row) and SBA (middle and bottom rows) for a patient with a clinical diagnosis of minimally-conscious state (maximum displacement 20.3 mm, 16/200 outlier volumes); red crosses denote inability to identify DMN activity in any of the 20 components extracted by ICA.** Due to the gross anatomical damage visible on the volumetric *T*_1_ scan, SBA with the right precuneus seed was not performed. Here, elevating preprocessing grade (left to right, P1–P5) revealed coherent activity between the left precuneus and angular gyrus: applying procedures 4 and 5, ICA decomposition became able to orthogonalize activity for this preserved DMN subset, and SBA maps were “cleaned” of unspecific physiological fluctuations that originally extended to areas of gross anatomical damage.

**Figure 9:**
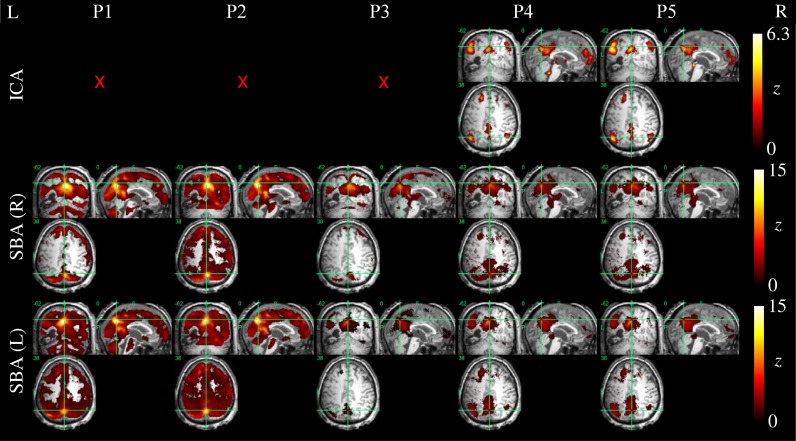
**DMN functional connectivity maps computed with ICA (top row) and SBA (middle and bottom rows) for a patient with a clinical diagnosis of minimally-conscious state (maximum movement 5.0 mm, 24/200 outlier volumes); red crosses denote inability to identify DMN activity in any of the 20 components extracted by ICA.** Here, applying procedures 4 and 5 made ICA decomposition capable of revealing coherent activity between the precuneus, angular gyri and a cluster in the left superior frontal lobe. For SBA, a non-monotonic effect is evident, whereby applying procedure 3 removed substantial unspecific covariation across the brain parenchyma, and the subsequent steps implemented in procedures 4 and 5 revealed coherent activity between the precuneus, angular gyrus and superior frontal lobe.

Example ICA and SBA maps from representative cases are shown in Figures 6–9. Mirroring the numerical results reported in Table 2, ICA and SBA demonstrated a markedly different response to preprocessing grade. For ICA, improving filtering progressively enhanced the extent and intensity of correlation in the DMN nodes whereas for SBA, a gradual attenuation of diffuse, unspecific activity was observed. Reassuringly, as preprocessing was refined the results of ICA and SBA tended to converge. The entity of the effect of preprocessing at first could appear substantially larger for SBA than ICA, but one should consider that ICA failed to extract an identifiable DMN component in Figures 7–9 unless procedure P3 or higher was utilized. Notably, in Figure 7 an apparent “hemispheric splitting” of DMN activity is visible: the occurrence of this effect increased with preprocessing grade (i.e., P1: 2, P2: 5, P3: 6, P4: 9, and P5: 10 patients).

## Discussion

The present study extends previous comparative evaluations of rs-fMRI preprocessing (e.g., Murphy et al., 2009; Weissenbacher et al., 2009; Van Dijk et al., 2010) to the specific population of patients in vegetative and minimally conscious state, which presents particular challenges related to substantial head movements and extensive anatomical damage. In this group, detection of residual neuroelectric activity is crucial for understanding disease staging and progression and the effect of possible rehabilitation therapies, and if rs-fMRI is performed to support clinical decision-making false negatives may have severe consequences; on the contrary, detection of DMN false-positives is less plausible. While deep sedation and even general anesthesia are routinely used for structural imaging in DoC patients, they would introduce severe confounds in rs-fMRI studies as they unavoidably depress central neural activity (e.g., Greicius et al., 2008; Boveroux et al., 2010; Deshpande et al., 2010; Stamatakis et al., 2010). Light sedation affects DMN activity more mildly but is generally insufficient to completely avoid movement (Giacino et al., 2006; Greicius et al., 2008).

As in Weissenbacher et al. (2009) the removal of co-variance with the movement vectors and average parenchyma signal coupled with BPF was found to have a substantial impact on the generation of DMN functional connectivity maps by both ICA and SBA. Here, additional steps to reject volumes contaminated by gross movement artifacts and to mask the ventricles were inserted and found to further improve data quality (Tables 2–4). The DMN maps generated with the two techniques showed a complementary sensitivity to preprocessing grade (i.e., procedures P1 to P5). For ICA, improving preprocessing resulted in larger and more significant correlations (Figures 2, 3, Tables 2, 3), with several cases in which an identifiable DMN component could not be extracted at all from data preprocessed with the most basic procedures (examples in Figures 7–9). By contrast, SBA maps initially displayed severe contamination by diffuse, unspecific correlations due to physiological noise and appeared progressively cleaner, with less significant but more focal and well-defined correlations for advanced preprocessing procedures (Figures 3, 4). In line with previous reports, ICA was considerably less sensitive to the choice of preprocessing steps than SBA (Weissenbacher et al., 2009; Van Dijk et al., 2010; Power et al., 2012), especially in terms of separation of DMN activity from spurious correlations in other brain areas (Figure 4 and examples in Figures 6–9) but it was clearly not indifferent. As indicated in Tables 2, 3, ICA appeared relatively more sensitive to movement variance removal and BPF (P2), whereas SBA was most heavily influenced by removal of unspecific temporal variance (P3).

An important element of the proposed preprocessing chain is the automated removal of volumes contaminated by gross movement artifacts, which can be automatically identified as outliers on the basis of the residual root mean square intensity difference calculated during rigid-body realignment. This approach appears particularly convenient as it is completely operator-independent and straightforward to implement, with minimal assumptions on the type of artifacts Carp (2013). As indicated in Figure 1, it is important to reject any contaminated volumes prior to performing further preprocessing steps, namely slice-timing correction and temporal filtering, which entail assumptions on the relationships between consecutive time-points. The proposed “two-tier” approach, involving replacement by interpolation unless the number of contaminated volumes is excessive, has specific advantages in terms of minimizing the occurrence of undesirable temporal discontinuities (e.g., Carp, 2013). *Per-se*, the connectivity inferences drawn by both ICA and SBA are intrinsically insensitive to the removal and linear interpolation of time-points just as they are to temporal aliasing (Van Dijk et al., 2010).

In addition to removing spurious signal sources, the rejection of movement outliers also improves the proportion of real movement-related variance that can be removed through multilinear regression with respect to the movement vectors. This is a particularly important benefit, because in the presence of outliers the linear regression may be dominated by abnormally large or small signal levels for some volumes and thereby fail to properly capture the covariance with real movement. Movement can have substantial and highly region-dependent effects on the BOLD time-courses, as well-typified by the strong spectral correlations observed by Soddu et al. (2012) between the movement vectors and BOLD activity in a brain death patient.

Because DoC patients can present severely enlarged ventricles, due to increased intracranial pressure as well as atrophy, there is the possibility that the signal fluctuations due to pulsatile cerebrospinal fluid flow may be substantially over-represented with respect to a healthy brain, biasing the determination of the ICA un-mixing matrix (e.g., Power et al., 2012) and impairing the detection of weaker neuronal sources and affecting SBA through contamination of the seed signals by partial voluming with pulsating fluid in ventricles and sulci. Here, the effect of introducing the rejection of outlier volumes (procedure P4) and ventricle masking (procedure P5) was more limited in comparison to that of BPF and removal of movement and global variance (procedures P2 and P3), yet at group level it was statistically significant for SBA, particularly reflecting into reduced extent of spurious correlations outside the expected DMN nodes. Importantly, even though for ICA the effect of these additional steps was not statistically significant at group level (Table 3), in several patients (e.g., Table 4 and Figures 7–9) ICA failed to extract an identifiable DMN component for procedures P1–P3 but not P4–P5, and in specific cases (e.g., Figure 6) the visual appearance of activity in DMN nodes improved appreciably for procedure P5. Since procedures P4 and P5 are computationally parsimonious, we advise that they are always included in the preprocessing pipeline. While it may be argued that ICA should in principle not need any preprocessing thanks to its ability to isolate independent components, our data confirm that careful preprocessing is important not only for SBA, but also for ICA, as it improves component un-mixing and therefore reduces the risk of false negatives in DMN detection; of note, this effect was evident in patients but not in controls, plausibly reflecting differences in the entity of movement artifacts and strength of component signals.

Further insight into the effect of movement on correlations between regional time-courses is provided by the linear regression analyses. As discussed in Power et al. (2012) and Satterthwaite et al. (2013), correlation between two regions can be inflated if they undergo a common translation or masked if they undergo a rotation around a point located between them. In other words, head movement artifactually increases functional coupling across local networks and decreases it for long-range connections (Power et al., 2012; Van Dijk et al., 2012). Here, elevating preprocessing grade had markedly different effects on the evaluation of the shorter latero-lateral connections between the lateral parietal cortex and the precuneus and longer anterior-posterior connections between the precuneus and the medial prefrontal cortex (Figure 5). In the first case, a gradual reduction of the correlation coefficients was observed, signaling that a substantial part of the raw covariance was induced by movement and global fluctuations. In the second case, a biphasic response emerged: while the correlation coefficients overall increased, initially regressing-out movement-related variance (procedure P2) boosted the correlations but subsequently eliminating global variance reduced them again (procedure P3). This suggests that movement initially masked the covariance between these regions, which was, however, dominated by unspecific, global fluctuations in patients. In controls, the effect of preprocessing procedure on latero-lateral connections was more constrained, plausibly due to less movement, but greater changes were observed in anterior-posterior connectivity with respect to patients: this plausibly reflects the fact that frontal DMN connectivity is strong in controls but very weak or lost in patients, and may also be related to different movement patterns along the three axes. These different effects along the lateral and anterior-posterior directions agree with previous investigations and further underline the potential for complex confounding effects (Power et al., 2012; Van Dijk et al., 2012; Satterthwaite et al., 2013), stressing the importance of adopting comprehensive preprocessing approaches that attempt to eliminate as much spurious signal variance as possible.

The present study has several limitations that need to be considered. First, because no gold-standard reference for DMN activity is available, the evaluation necessarily remains an empirical one, and it is not possible to formally confirm how much of the signal variance eliminated in each step was artifactual rather than neural. Yet, since the DMN has a highly stereotyped appearance (i.e., is expected to involve specific nodes at relatively stable anatomical locations), its increased detectability reassured on the overall beneficial effect of the suggested preprocessing techniques (Esposito et al., 2008). Second, almost all DoC patients are characterized by extensive brain anatomical abnormalities, and the present study did not consider in detail the effect of imperfect normalization caused by poor structural similarity between the damaged individual brains and the standardized healthy brain template. This issue, which equally affects all other studies in this area, will need to be evaluated in future studies. Third, anti-correlations were not considered and the evaluation of component detectability with ICA and SBA was limited, as in some other studies in this area, to positive correlations. While there is increasing evidence that negative correlations may also represent architecturally important forms of functional connectivity, the interpretation of such effects remains unclear, hence they were not considered here (e.g., Rosazza and Minati, 2011). Fourth, we did not include relative displacements in our regressors as advised by Satterthwaite et al. (2013) and Power et al. (2012); this parameter needs to be considered in future work. Our approach otherwise maps closely with their suggestions and includes additional steps that are specifically beneficial in this population where movement is substantial and requires outright rejection of volumes and the extent of atrophy and weak signal justify the masking of non-brain structures. Fifth, the DMN component was selected and rated manually by expert operators. Spatial templates of the DMN (Esposito et al., 2008) are available, as well as automated techniques based on multi-dimensional “fingerprints” and support vector machines which have been successfully applied to data from DoC patients (Soddu et al., 2012), and the effect of preprocessing choices on their performance should be evaluated in future work (De Martino et al., 2007). An inherent issue is the inability to quantify the proportion of false negatives: because no gold-standard of DMN integrity exists, our data suggest that elevating preprocessing grade reduces the incidence of false negatives, but do not enable quantifying the risk of false negatives. That parameter will need to be determined in future studies addressing correlation with clinical status as well as test-retest reliability. Finally, recent work has demonstrated the possibility to explicitly extract the cardiac and respiratory regressors directly from the fMRI data (Beall, 2010), and additionally offered specific advice regarding movement regressor filtering (Hallquist et al., 2013) and rejection of movement-contaminated data (Christodoulou et al., 2013); the corresponding techniques will need to be considered to update and extend the results obtained in the present investigation.

## Conclusion

This study provides a comprehensive evaluation of the effect of data preprocessing choices on rs-fMRI in DoC patients, and corroborates the existing literature in this area through systematic comparison of five preprocessing procedures of increasing complexity. ICA and SBA were both found to be significantly impacted by data preprocessing settings, albeit with different patterns. Elevating preprocessing grade improved the ability of ICA to successfully un-mix DMN activity and generally enhanced the significance and extent of DMN correlations. By contrast, for SBA high-grade preprocessing had the principal effect of reducing contamination by unspecific, systemic signal sources, reflecting in progressively more focal and well-defined activity patterns. As preprocessing grade was elevated, the topographical maps provided by the two techniques tended to converge. The results strongly underline the importance of performing high-grade preprocessing, including rejection of outlier volumes, ventricle masking, removal of movement related and global signal covariance and BPF. Even though a gold-standard measure of connectivity preservation does not exist, since the DMN has highly characteristic topographical features the observation that its detectability increases with better preprocessing indicates reduced risk of false negative errors. We propose that the described preprocessing procedures should be adopted as a minimum standard in future studied in this area to reduce the probability of wrongly inferring that DMN activity is absent, with potential implications for clinical management.

### Conflict of interest statement

The authors declare that the research was conducted in the absence of any commercial or financial relationships that could be construed as a potential conflict of interest.
