# Corrigendum: Impact of functional MRI data preprocessing pipeline on default-mode network detectability in patients with disorders of consciousness

**DOI:** 10.3389/fninf.2014.00050

**Published:** 2014-05-16

**Authors:** Adrian S. Andronache, Cristina Rosazza, Davide Sattin, Matilde Leonardi, Ludovico D'Incerti, Ludovico Minati

**Affiliations:** ^1^Neuroradiology Unit, Fondazione IRCCS Istituto Neurologico “Carlo Besta”Milano, Italy; ^2^Scientific Department, Fondazione IRCCS Istituto Neurologico “Carlo Besta”Milano, Italy; ^3^Neurology, Public Health, Disability Unit, Scientific Department, Fondazione IRCCS Istituto Neurologico “Carlo Besta”Milan, Italy

**Keywords:** functional MRI (fMRI), resting-state, functional connectivity, disorders of consciousness, vegetative state, minimally-conscious state, data preprocessing

The authors of the article by Andronache et al. apologize that the funding acknowledgment “*The Start-up Coma Research Centre (CRC) project was also supported by the European Foundation for Biomedical Research (FERB)*” was omitted from the original publication.

## Conflict of interest statement

The authors declare that the research was conducted in the absence of any commercial or financial relationships that could be construed as a potential conflict of interest.
